# Computational mechanism underlying switching of motor actions

**DOI:** 10.1371/journal.pcbi.1012811

**Published:** 2025-02-10

**Authors:** Shan Zhong, Nader Pouratian, Vassilios Christopoulos

**Affiliations:** 1 Neuroscience Graduate Program, University of California Riverside, Riverside, California, United States of America; 2 Alfred E. Mann Department of Biomedical Engineering, University of Southern California, Los Angeles, California, United States of America; 3 Department of Neurological Surgery, UT Southwestern Medical Center, Dallas, Texas, United States of America; 4 Department of Bioengineering, University of California Riverside, Riverside, California, United States of America; University of Virginia, UNITED STATES OF AMERICA

## Abstract

Survival of species in an ever-changing environment requires a flexibility that extends beyond merely selecting the most appropriate actions. It also involves readiness to stop or switch actions in response to environmental changes. Although considerable research has been devoted to understanding how the brain switches actions, the computations underlying the switching process and how it relates to the selecting and stopping processes remain elusive. A normative theory suggests that switching is simply an extension of the stopping process, during which a current action is first inhibited by an independent pause mechanism before a new action is generated. This theory was challenged by the affordance competition hypothesis, according to which the switching process is implemented through a competition between the current and new actions, without engaging an independent pause mechanism. To delineate the computations underlying these action regulation functions, we utilized a neurocomputational theory that models the process of selecting, stopping and switching reaching movements. We tested the model predictions in healthy individuals who performed reaches in dynamic and uncertain environments that often required stopping and switching actions. Our findings suggest that unlike the stopping process, switching does not necessitate a proactive pause mechanism to delay movement initiation. Hence, the switching and stopping processes seem to be implemented by different mechanisms at the planning phase of the reaching movement. However, once the reaching movement has been initiated, the switching process seems to involve an independent pause mechanism if the new target location is unknown prior to movement initiation. These findings offer a new understanding of the computations underlying action switching, contribute valuable insights into the fundamental neuroscientific mechanisms of action regulation, and open new avenues for future neurophysiological investigations.

## Author summary

Humans exhibit a remarkable ability to regulate their actions in response to changing environmental demands. An essential aspect of action regulation is action inhibition that occurs when stopping unwanted or inappropriate actions. However, everyday life rarely calls for complete inhibition of responses without switching behavior to adapt to new situations. Despite extensive research to understand how the brain switches actions, the computations underlying the switching process and how it relates to the selecting and stopping processes remain elusive. Part of this challenge lies in the fact that these processes are rarely studied together, making it difficult to develop a unified theory that explains the computational aspects of the action regulation mechanism. The current study aims to delineate the computations underlying action regulation functions that involve inhibitory control, explore how these functions interrelate, and how they can be implemented within brain networks, opening new avenues for future neurophysiological investigations.

## Introduction

Operating effectively in an uncertain and dynamic environment requires not only the ability to accurately prepare and perform actions, but also the flexibility to inhibit actions in response to environmental changes. When driving on a city road, we often need to stop at red lights, crosswalks or intersections. However, everyday life rarely calls for complete stopping of actions without subsequent behavioral adjustments. In fact, altering environmental conditions frequently leads us to abandon planned or ongoing actions followed by a switching behavior to adapt to new situations. Understanding the mechanisms of selecting, stopping and switching actions is important for revealing how the brain functions in a variable and evolving environment.

The current study aims to dissociate the mechanisms involved in stopping and switching reaching movements. Normative theories suggest that a current action must first be inhibited by an independent inhibitory mechanism (i.e., pause mechanism) before switching to a new action [1,2]. This view computationally conceptualizes response inhibition as a race between two “runners” (i.e., processes) - a “go runner” initiated by the presentation of external stimuli and a “stop runner” triggered by a stop signal. If the stop runner wins the competition, the response is inhibited. Otherwise, the response is emitted [2–4]. Therefore, when switching action is required, the current go process must first be interrupted by the stop process before another go process generates a new action. This theory has received significant support from neurophysiological and functional neuroimaging studies that have identified the basal ganglia (BG), and in particular the subthalamic nucleus (STN), as a key region in canceling an already selected, or currently performed, action when goals change [5–7]. Overall, these studies suggest that switching actions involves the same processes as stopping actions, with the only difference being that a new action is generated after the old one is suppressed.

However, neurophysiological recordings in non-human primates (NHPs) challenged this “go-stop-go” theory, suggesting that an independent pause mechanism may not be required when switching actions [8]. Instead, switching actions can be implemented through a competition process between the current and the new action without engaging the pause circuitry. This theory is considered an extension of the “affordance competition hypothesis” according to which multiple motor actions are formed concurrently and compete over time until one has sufficient evidence to win the competition [9–11]. This hypothesis predicts that action selection is made through a competition within the same circuit that plans and produces the actions themselves. It predicts that the same neurons involved in the initial action selection process remain active in adjusting and even switching between actions during overt behavior [8].

Therefore, the main question is whether the switching process is an extension of the stopping process or it can be implemented through a different mechanism. To address this question, we trained healthy young adult participants to perform reaching movements with a 2D joystick to either a single target or one selected from two targets assigned with different expected rewards. In a subset of trials, participants had to completely stop or switch their reaching movements. To better elucidate the action regulation mechanisms in selecting, stopping and switching of actions, we modeled the reaching tasks within a neurodynamical computational framework that combines dynamic neural field (DNF) theory [12,13] with stochastic optimal control (SOC) theory [14,15]. The framework was recently developed to simulate motor behavior and the underpinning neural mechanisms in a variety of visuomotor tasks that occur in dynamic and uncertain environments [16,17]. By modeling the experimental tasks within the neurocomputational framework, we provide evidence that reaching planning does not involve a proactive pause mechanism, unless a stop signal is anticipated. Interestingly, the mechanism for switching ongoing actions depends on whether the new target location is known prior to the switch signal. The participants exhibited slower reaction times for switching actions when they were aware of the new target location prior to the switch signal, compared to when they were unaware of it. This suggests that when the new target location is unknown, an independent pause mechanism may be engaged to suppress the ongoing action. Conversely, when the new target location is known, action switching may occur without activating the pause mechanism, indicating a different implementation pathway. Overall, our study provides a putative model for the intricate processes involved in stopping and switching actions, opening new avenues to better understand how the brain regulates actions in dynamic and uncertain environments.

## Results

### Experimental paradigms

**Fig 1:**
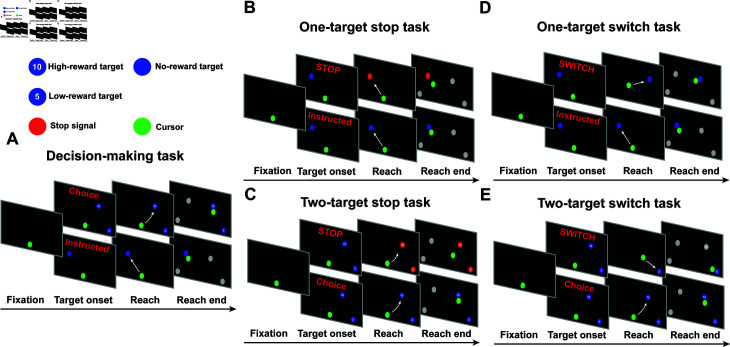
Experimental setup for the decision-making task, the stop signal task and the switch task. (A) Experimental setup for the decision-making task, including instructed trials and choice trials. (B) Experimental setup for the one-target stop task, including stop trials and instructed trials. (C) Experimental setup for the two-target stop task, including stop trials and choice trials. (D) Experimental setup for the one-target switch task, including switch trials and instructed trials. (E) Experimental setup for the two-target switch task, including switch trials and choice trials.

Participants were instructed to perform rapid reaching movements using a 2D joystick in 3 experimental tasks: decision-making (i.e., action selection), stop signal (i.e., outright stopping) and switch task (Fig 1). The decision-making task includes choice trials, during which participants had to decide between two targets (blue circles) associated with different expected rewards that were presented either on the same or opposite visual fields (Fig 1A). Choice trials were interleaved with instructed trials, in which only one single target was presented in the field. The stop signal task is similar to the decision-making task with the difference that in a random subset of trials (i.e., 33*%*), the color of the target(s) turned red after a short variable delay (stop signal delay, SSD), indicating that the participants needed to immediately stop any planned/ongoing reaching movements. Stop trials occur both in instructed (Fig 1B) and choice trials (Fig 1C). If the participants successfully stopped their action, the SSD increased by 50 ms, making the next stop trial more challenging, otherwise the SSD decreased by 50 ms, making the next stop trial easier. Finally, the switch task is also similar to the decision-making task with the difference that in a random subset of instructed trials (i.e., 33*%*), the original target was replaced after a short variable delay (switch signal delay, SWSD) by a second target, prompting the participants to switch their actions towards the new target location (Fig 1D). Similarly, in another 33*%* random subset of choice trials, the high-reward target disappeared, and the participants had to move to the remaining one (i.e., low-reward target) (Fig 1E). If the participants successfully switched their actions without crossing the old target location, the SWSD increased by 50 ms, making the next switch trial more challenging. Otherwise, SWSD decreased by 50 ms, making the next switch trial easier.

**Fig 2:**
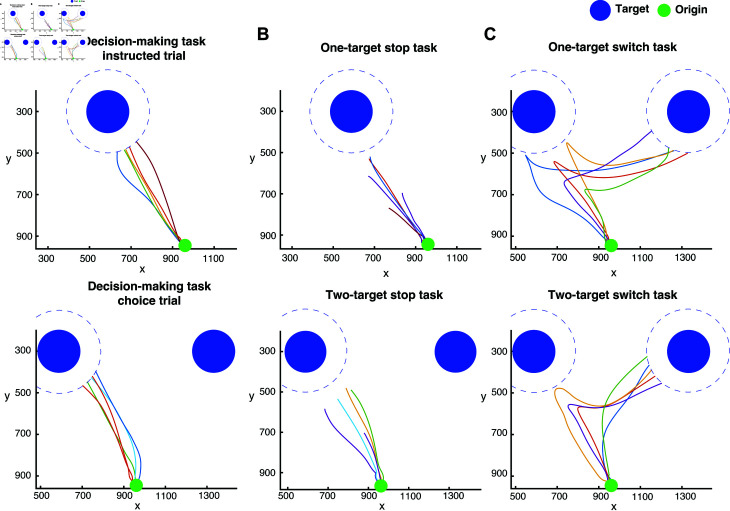
Example human reaching trajectories in the decision-making, stop signal and switch tasks. (A) Five sample trajectories, all starting from the origin (green dot) and reaching either the instructed target (instructed trial, top) or the selected target (choice trial, bottom). The targets are shown in blue filled circles. Note that the target disappears as soon as the edge of the cursor touches the edge of the target. Since we tracked the center of the cursor, the trajectories end slightly before reaching the target, as indicated by the blue dashed circles. (B) Five sample trajectories from successful stop trials in both the one-target stop task (top) and the two-target stop task (bottom). In these cases, the trajectories end further from the target compared to panel A, showing that the participants did not reach the targets. (C) Five sample trajectories from successful switch trials in the one-target switch task (top) and the two-target switch task (bottom). In the one-target switch trial, the initial target on the left disappears, and a new target appears on the right. In the two-target switch trial, the target with the higher reward (on the left) disappears, leaving the lower-reward target on the right.

**Fig 3:**
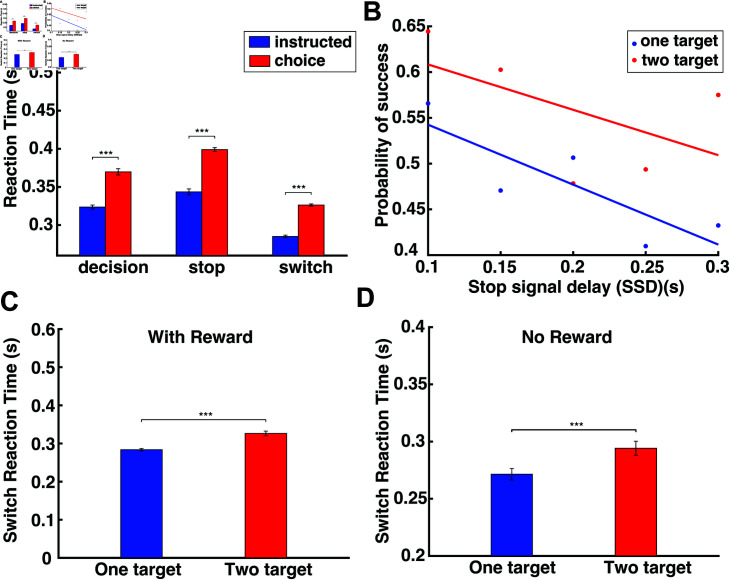
Human performance in the decision-making task, the stop-signal task and the switch task. (A) Bar plots of the RT in the instructed trials (blue) and choice trials (red) of the decision-making task, the stop-signal task and the switch task. (B) The probability to successfully stop an action as a function of SSD for one-target (blue) and two-target (red) stop tasks. (C) Bar plots of the SRT for the one-target and two-target switch tasks (with reward). (D) Bar plots of the SRT for the one-target and two-target switch tasks (no reward). Error bars correspond to SE.

### Motor strategy and performance in selecting, stopping and switching of actions

The participants generated highly stereotyped reaching movements when they were instructed to reach towards a target location (Fig 2A top panel) or when they were free to choose between two targets presented simultaneously on the screen (Fig 2A bottom panel). They were also capable of stopping or switching their movements when they were prompted both in instructed (Fig 2B and2C, top panels) and choice trials (Fig 2B and2C, bottom panels). We computed the reaction time (RT) in the instructed and choice trials as an index of motor preparation of the reaching movements. The RT was computed as the time interval between the presentation of the target(s) on the screen and response initiation.Fig 3A illustrates the average RT across all trials and participants for instructed and choice reaches in the three experimental paradigms. A two-way ANOVA revealed statistically significant differences in RT in the experimental tasks ($p<10^{-6}$) and type of movements (i.e., instructed vs. choice) ($p<10^{-6}$). A post-hoc multiple-comparison analysis using the Tukey test indicated that instructed trials had shorter RTs than choice trials in all three experimental tasks ($p<10^{-7}$). Furthermore, RT was longer both in instructed and choice trials when participants expected a stop signal compared to trials in which they did not anticipate to stop their actions (i.e., decision-making task) ($p<10^{-7}$). These findings are consistent with the results from our previous study [17] in which we reported longer RTs when anticipating a stop signal in instructed trials. Notably, we found that participants did not exhibit proactive inhibition in switch tasks, that is, they did not prolong their response when they anticipated to switch their actions both in instructed and choice trials (Fig 3A). In fact, RT was shorter in the switch task compared to the decision-making task both in instructed and choice trials ($p<10^{-7}$), even though participants were given an extra 1.0 s to complete a trial if a switch signal was shown. These results suggest that participants do not take a proactive action for slowing down their movement initiation when a switch signal is anticipated. The shorter RT in the instructed and choice trials of the switch task compared to the decision-making task can reflect an adequate amount of practicing reaching movements - i.e., decision-making task was performed before the switch task. In our previous study [17], the decision-making task was also performed before the stop signal task, yet both neurotypical participants and Parkinson’s disease (PD) patients still exhibited longer RTs when they anticipated a stop signal, which further supports the notion that participants do not adopt a proactive inhibitory behavior when anticipating a switch signal.

We also evaluated the performance of the participants by computing the probability to successfully stop an action for different SSD values both in one-target and two-target stop tasks. We found that the probability to stop an action is inversely correlated with SSD - i.e., the longer the SSD, the lower the probability to completely suppress a reaching movement on time - both in one-target and two-target trials (Fig 3B). Interestingly, the probability of successfully stopping an action was higher when participants were free to choose between two targets (i.e., two-target stop trials) than when they were instructed to reach towards a single target location (i.e., one-target stop trials).

Finally, we computed the time it takes for the participants to respond to a switch signal (switch reaction time, SRT) in instructed and choice trials. The results showed that participants had a shorter switch response when they were instructed to move towards a single target location than when they had to choose between two target locations - i.e., SRT is shorter in one-target switch task than in the two-target switch task (Fig 3C, two sample t-test analysis,*p* < 0 . 001). One potential explanation is that participants became less motivated when they were prompted to switch their reaching movements from a high- to low-reward targets, since motivation is correlated with expected reward [18,19]. To assess whether the longer SRT is due to the reduction of the expected reward, we recruited 6 participants to perform a modified version of the switch task. The modified switch task was similar to the switch task described above, with the only difference that the targets were not assigned with an expected reward, and the switching occurred once the reach trajectory exceeded a random distance threshold. The results showed that SRT was still longer for two-target switch trials compared to one-target switch trials (Fig 3D, two sample t-test analysis,*p* < 0 . 001). Therefore, we can conclude that people had slower responses to switch their actions when they were aware of the new target location prior to movement initiation (i.e., choice trials) than when they were not aware of the new target location (i.e., instructed trials).

### Predicting motor behavior in action regulation tasks using a neurocomputational theory

To better understand the computations of selecting, stopping and switching actions, we modeled the three experimental tasks within a recently developed neurocomputational theory which models action regulation functions that involve motor inhibition [17]. The neurocomputational theory combines DNF theory and SOC theory and includes circuitry for perception, expected outcome, effort cost, stop signal, pause mechanism, action planning and execution. It is based on the affordance competition hypothesis, in which multiple actions are formed concurrently and compete over time until one has sufficient evidence to win the competition [9,20,21]. In a recent study, we showed that the theory can predict many key aspects of motor behavior in motor tasks that involve selecting and stopping of actions, including spatial characteristics of the reaching trajectories, as well as reaction time, movement velocity, probability of successfully stopping actions, and so on [17]. The architectural organization of the framework is shown inFig 4. Each DNF simulates the dynamic evolution of firing rate activity of a network of neurons over a continuous space with local excitation and surround inhibition. The core component of the framework is the “reach planning” field that has two roles: i) activating downstream stochastic optimal controllers that generate actions towards particular directions and ii) integrating information from disparate sources associated with actions, goals, and contextual requirements into a single value (normalized neural activity) that characterizes the relative desirability (i.e., “attractiveness”) of the active actions. The reach planning field receives excitatory input (green arrows) from the “spatial sensory input” field (which encodes the angular representation of the targets in an egocentric reference frame) and “expected outcome” field (which encodes the rewards associated with moving towards particular directions), as well as inhibitory input (red arrows) from the “reach cost” field (encodes the effort required to move towards particular directions) and the “pause” field, which suppresses ongoing (or planned) actions when motor inhibition is required. Each neuron in the reach planning field is connected with a control scheme that generates reaches. Once the activity of a neuron exceeds an action initiation threshold, a decision is made, and the corresponding controller is triggered and generates a sequence of motor actions towards the preferred direction of that neuron (more details are presented in Materials and Methods section and our recent study [17]).

**Fig 4:**
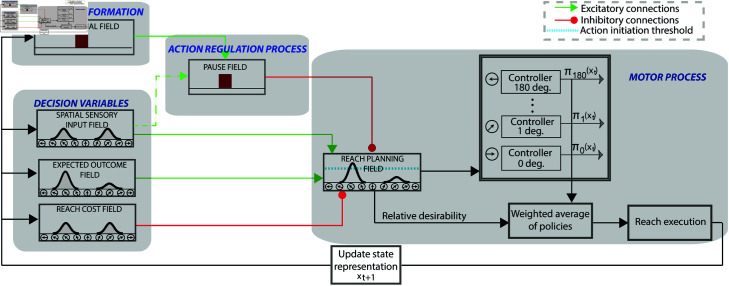
Model Architecture. The reach planning field encodes the planned direction of arm movement in an egocentric reference frame. It integrates sensory information, expected reward and cost, as well as inhibition from the pause field, to generate the “relative desirability”, or the attractiveness of an action policy with respect to alternatives. The relative desirability was used as the weight to compute the final action policy, and a reaching movement is generated, moving the hand towards the selected direction.

In the current study, we extended the theory to model the computations involved in switching actions. We considered the following potential architectures that can implement the pause mechanism in action regulation tasks.

- Architecture 1:*The pause mechanism is involved in both stopping and switching actions*

The pause mechanism is engaged anytime that an action has to be suppressed. In the stop signal task, the pause field is activated during action planning, and further activated to completely suppress all ongoing/planned actions following a stop signal. Similarly, in the switch task, the pause field is also activated during action planning, and further engaged to suppress the ongoing/planned actions before the new action is performed to implement the change of action plan.

- Architecture 2:*The pause mechanism is involved only in outright stopping of actions*

An alternative architecture suggests that the pause mechanism is involved only in outright stopping of actions. On the contrary, the pause field is not activated during action planning, and switching to new actions is exclusively implemented within the reach planning field – i.e., switching of actions is achieved through a competition within the same circuit that guides the actions themselves. The very similar neurons in the reach planning field that guide action selection will continue to update their activities in the presence of new incoming information to switch the action when needed.

### Modeling reach decisions

**Fig 5:**
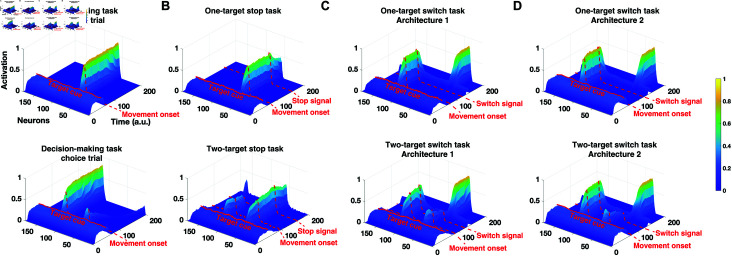
Simulated reach planning field neuronal activity changes in the decision-making task, the stop-signal task and the switch task. Simulated activity of 181 neurons from the reach planning field during: (A) Decision-making task (instructed trial and choice trial), (B) Stop signal task (one-target stop trial and two target stop trial), (C) Switch task under architecture 1 (one-target switch trial and two-target switch trial), and (D) Switch task under architecture 2 (one-target switch trial and two-target switch trial).

We modeled the decision-making task within the neurocomputational framework for both instructed and choice trials.Fig 5A depicts the simulated neural activity in the reach planning field for typical instructed (top panel) and choice (bottom panel) reaches, respectively. The activity starts at the baseline (resting state) before the target(s) are presented in the field. After the instructed target is presented, the activity of the single neuronal population tuned to the direction of the target increases. Once the activity exceeds the action initiation threshold, a reaching movement is generated towards the target location. In the choice trial, two neuronal populations selective for the targets start competing for selection via mutual inhibitory interactions. This competition leads to a longer RT in movement initiation in choice trials compared to instructed trials. Since the reach planning field receives excitatory inputs from the expected outcome field, the target reward influences choice preferences by shifting the selection bias towards the higher valued target - i.e., the activity of the neuronal population tuned to the higher valued target increases significantly compared to the neural activity associated with the lower valued target. Once the activity of a neuronal population exceeds the action initiation threshold, the competition is resolved and a reaching movement is initiated towards the selected target location.

### Modeling outright stopping of actions

We also modeled the stop signal task within the neurocomputational framework for both one-target and two-target trials.Fig 5B illustrates the simulated neural activity of the reach planning field for typical instructed (top panel) and choice (bottom panel) reaches that are prompted to completely stop a few time steps after departing from the origin. Note that the pause field is partially activated even before initiating an instructed or a choice reaching movement. This is based on human findings that reaches have longer RTs when participants anticipate a stop signal than when no stop signal is expected (response delay effect, RDE) (Fig 3, see also [17]). The inhibitory projection from the pause field leads to a reduction in the neuronal signal in the reach planning field when a stop signal is anticipated - i.e., the activity in the reach planning field is lower in the stop trials (Fig 5B) compared to the instructed and choice trials of the decision-making task (Fig 5A). Once the stop signal is cued, the activity of the pause field further increases to inhibit the activity of the reach planning field below the action initiation threshold, in order to completely stop the action (see also [17] for more details).

### Modeling switching of actions

Finally, we modeled the switch task within the neurocomputational framework for both one-target and two-target trials, considering two different architectures.Fig 5C depicts the simulated neural activity of the reach planning field in architecture 1 for typical instructed (top panel) and choice (bottom panel) reaches that are prompted to switch to a new target location a few time steps after departing from the origin. Similar to the stop-signal task, this architecture engages the pause mechanism during the planning phase of the reaching movement, and therefore the activity in the reach planning field is lower when switching is expected than when no switching is anticipated. Once the selected target is replaced by a second target, the activity of the pause field further increases to inhibit the current action while the new action is formed towards the new target location. Therefore, switching in this architecture is implemented by the pause mechanism and the mutual inhibitory competition between the current action and the new action. The reach planning field neural activity in the alternative architecture 2 is presented inFig 5D. In this scenario, the pause mechanism is not engaged during switching actions. Instead, the switching process is implemented by the same competition process responsible for generating the reaching movement to the initial target location - i.e., the neuronal population tuned to the new target inhibits the neuronal population tuned to the old target. In this case, the reaching neural activity prior to switching action is similar to the activity in tasks where no switching is anticipated.

### Simulated motor behavior in selecting, stopping and switching of actions

**Fig 6:**
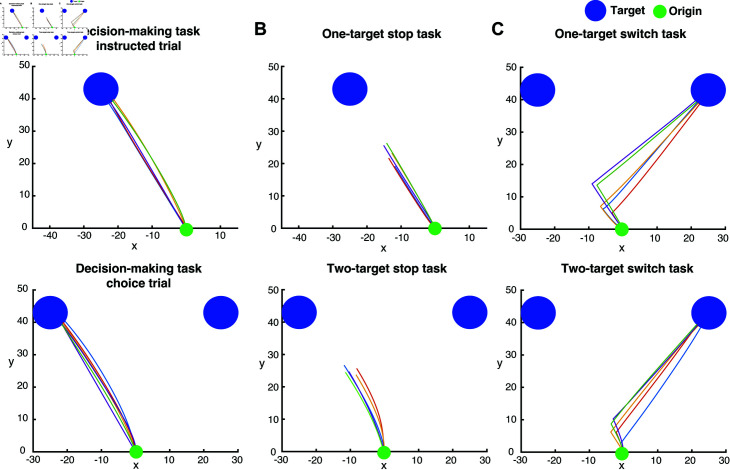
Example of simulated reaching trajectories in the decision-making task, the stop-signal task and the switch task. Simulated reaching trajectories starting from the origin (green dot) towards the blue target(s) in the (A) decision-making task, (B) stop signal task and (C) switch task. Note that the simulated cursor functioned as a point mass, therefore the trajectories ended at the target location in non-stop trials.

We simulated 100 decision-making trials with 50*%* instructed and 50*%* choice reaching movements. We also simulated 600 stop signal trials, consisting of an equal split between instructed and choice reaching movements, with 300 trials for each type. Out of these 600 trials, 500 were selected (250 from the instructed category and 250 from the choice category) to be prompted to stop after a short, variable delay (SSD). These 500 trials were diversified using 5 different SSDs, with 50 trials conducted for each SSD. Note that we simulated such a large number of reach trajectories in the stop signal task, so that we explored whether the model can predict the effects of the SSD and the number of targets on the probability of successfully stopping an action. Finally, we simulated 200 trials with 50*%* instructed and 50*%* choice reaching movements, but 100 of the trials (50 instructed and 50 choice trials) were prompted to switch the action after a short delay (SWSD). We performed one set of simulations with architecture 1 (i.e., the pause mechanism is involved in both stopping and switching actions) and another set of simulations with architecture 2 (i.e., the pause mechanism is involved only in outright stopping of actions).Fig 6 illustrates a sample of simulated reaching trajectories of the three experimental tasks (consistent across architectures 1 and 2), using only two targets located 60 degrees apart for the sake of simplicity.

**Fig 7:**
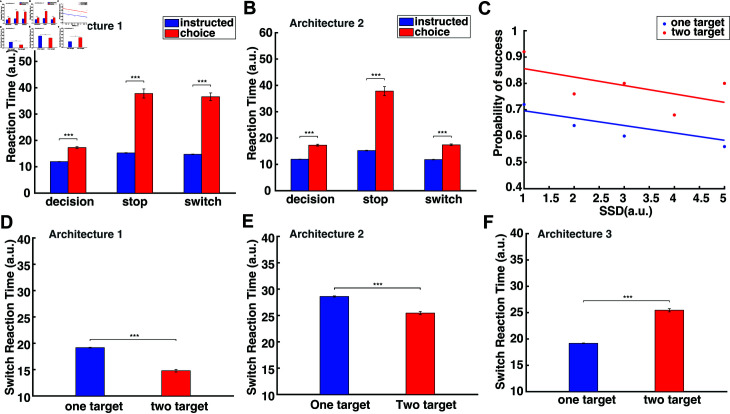
Simulated human behavior in the decision-making task, the stop-signal task and the switch task. (A) Bar plots of the simulated RT in the instructed and choice trials of the decision-making task, the stop signal task and the switch task under architecture 1. (B) Bar plots of the simulated RT in the instructed and choice trials of the decision-making task, the stop signal task and the switch task under architecture 2. (C) Simulated probability of successfully stopping a reaching movement as a function of SSD in one-target stop trials (blue) and two-target stop trials (red). (D) Bar plots of the simulated SRT for the one-target and two-target switch trials under architecture 1. (E) Bar plots of the simulated SRT for the one-target and two-target switch trials under architecture 2. (F) Bar plots of the simulated SRT for the one-target and two-target switch trials under architecture 3. Error bars correspond to SE. Architecture 1: The pause field is involved in the switching process. Architecture 2: The pause field is NOT involved in the switching process. Architecture 3: The pause field is involved in the switching process in the one-target condition, but not involved in the two-target condition.

#### Reaction time of simulated reaches.

Fig 7A and7B depict the average RT for the simulated instructed (blue) and choice (red) reaches in the three experimental tasks with (panel A, architecture 1) and without (panel B, architecture 2) the involvement of the pause mechanism in the switch task, respectively. A two-way ANOVA revealed statistically significant differences in RT in the experimental tasks (*p* < 0 . 001) and type of movements (i.e., instructed vs. choice) (*p* < 0 . 001) for both architectures. A post-hoc multiple-comparisons analysis using the Tukey test showed that choice reaches have a longer RT than instructed reaches (*p* < 0 . 001) in all three tasks for both architectures due to the inhibitory action competition when two targets are presented. For the model architecture 1 which involves a pause mechanism for switching actions, no significant differences were observed in RT when anticipating a stop or switch signal, both in instructed (*p* = 0 . 959) and choice trials (*p* = 0 . 959) (Fig 7A). On the other hand, for model architecture 2 which does not involve a pause mechanism for switching actions, RT is longer in instructed (*p* < 0 . 001) and choice trials (*p* < 0 . 001) when a stop signal is anticipated compared to when a switch signal is anticipated (Fig 7B). Instead, switch trials have approximately the same RT as decision-making trials - i.e., when no switch signal is expected (*p* = 1 . 000). The human findings are consistent with the results from the model architecture 2, in which the pause mechanism is not engaged in switching actions during movement planning, since participants responded faster when a switch signal was anticipated than when a stop signal was expected.

#### Probability to successfully stop actions.

We also computed the probability to completely stop a reaching movement in the stop signal task by measuring the number of successful trials at any given SSD in both instructed and choice reaches. Consistent with human findings, the model predicted that the probability to successfully stop reaches is inversely related to SSD - i.e., the higher the SSD, the lower the probability to successfully stop a reaching movement. Notably, and consistent with human findings, the model predicted that the probability to stop an action is higher in the two-target stop trials than in the one-target stop trials for any given SSD (Fig 7C). The reason is that the activity of the reach neurons in the choice trials is weaker than in the instructed trials (Fig 5B top and bottom panel) due to inhibitory competition between the neuronal populations that are selective for the two targets. Therefore, the activity of the reach neurons is inhibited faster in the two-target stop trials than in the one-target stop trials, explaining why it is easier to stop an action when you are free to choose between competing targets than when you are instructed to move towards a particular target location.

**Fig 8:**
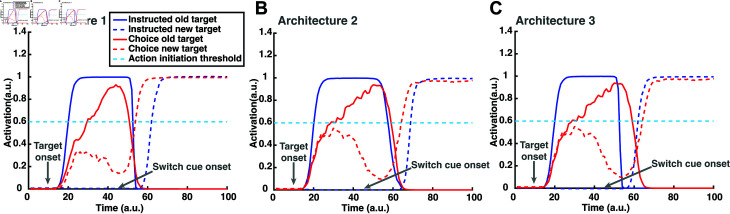
Reach planning field single neuronal activity during switch trials. Simulated reach planning field single neuronal activity changes during one-target and two-target switch trials under architectures 1(A), 2(B) and 3(C). Color code: Blue: one-target switch trial. Red: two-target switch trial.

#### Switch reaction time of simulated reaches.

Both model architectures predict that the reaction time for switching actions (SRT) will be longer in one-target trials than in two-target trials (Figs 7D and7E). The reason is that the reach neural activity associated with the non-selected target in the two-target trials is often not completely suppressed before the switching cue is given. Hence, the activity of the selected target is weaker in the two-target trials as compared to the one-target trials when reaching towards the same target location.Fig 8A and8B depict the simulated neural activity of a single reach neuron in an instructed trial (blue trace) and of two reach neurons, one from each population, in a choice trial (red trace) for architectures 1 and 2, respectively. Neurons are centered at the target location(s). Note that the neural activity associated with the new target location exceeds the action initiation threshold faster in the choice trial (red discontinuous trace) than in the instructed trial (blue discontinuous trace) in both architectures. Subsequently, switching of action in choice trials is more readily and quickly made than in instructed trials, because the neural representation of the new target location - the one that was not originally selected - has been formed prior to the switch signal. On the other hand, the neural representation of the new target location has not been formed prior to switch signal in the instructed trials, since the model has been instructed to generate movements towards one single target location without knowing the new target location.

Interestingly, model predictions contradict the human findings. Participants exhibited longer SRT in two-target switch trials than in one-target switch trials, both when they performed the reward-based and the non-reward-based (i.e., internally-guided) reaching tasks. One potential explanation is that one-target switch trials are governed by different switching mechanisms than the two-target trials during action execution. For instance, an alternative third architecture is that the pause mechanism is activated only in the instructed trials to inhibit the ongoing action. The reason is that the neural activity of the current action is too strong to be inhibited on-time by the new incoming action, which will be formed only after the switch signal. On the other hand, the neural activity of the selected target in the two-target trials is weaker compared to the one-target trials, due to the residual activity from the unselected target that inhibits the neural activity of the selected target. Therefore, the new incoming action, which is already formed before the switch signal, can inhibit the current action when needed without the contribution of the pause mechanism.Fig 8C illustrates the simulated neural activity of a single reach neuron in an instructed trial (blue trace) and of two reach neurons, one from each population, in a choice trial (red trace) under this model architecture. We simulated the switch task within the new architecture and found that the instructed trials have shorter SRT than the choice trials (Fig 7F). Taken together, our results suggest a third potential architecture (i.e., a combination of architectures 1 and 2) of the switching process, in which the pause mechanism is not active during action planning, but it is activated in action execution once a switch signal is detected, but only when reaches are instructed to a single target location.

## Discussion

### General

Humans can rapidly regulate actions according to evolving environmental demands. At the same time, impairments of action regulation have been identified across a number of neurological and psychiatric diseases, including PD, obsessive compulsive disorder (OCD), and Tourette syndrome [22–29]. Given the ubiquity of action regulation in everyday life, its critical role in survival, and its impairment across a variety of neurological and psychiatric diseases, understanding the mechanism of action regulation is of high value and impact. A key component of action regulation is action inhibition that occurs when stopping unwanted or inappropriate actions. Normative theories have also suggested that action inhibition plays a critical role in switching between actions in response to environmental changes. An ongoing (or planned) action has to be first inhibited, before switching to a new action [1,2]. A popular view is that when the pre-supplementary motor area (pre-SMA) detects the co-activation of different responses - a current response and a new response for switching action - it activates the STN to temporarily suppress the current response (reviewed by [30,31]). This role of the STN as a “pause mechanism” aligns with findings that neurons in the ventral STN rapidly activate in response to stop signals [7]. Thus, the ventral STN likely suppresses the ongoing action, enabling the generation of a new response. However, an alternative theory suggests that switching action might not always necessitate an independent action inhibition process (i.e., a pause mechanism). Instead, the same neurons within the motor areas that are involved in selecting the initial action will continue to be involved in adjusting and even switching actions during overt behavior[8,21]. This suggests that the brain may not always need to engage an independent pause mechanism to switch actions, particularly in scenarios where multiple potential actions are concurrently represented. Therefore, the mechanisms underlying how the brain selects, stops and switches between actions, as well as how these seemingly disparate functions inter-relate both behaviorally and computationally still remain elusive.

Our study focuses on the intricate interplay between action selection, stopping, and switching, and how these processes are interrelated within the human brain. We trained healthy young adult participants to perform reaching movements with a 2D joystick for running behavioral tasks that involve action selection, stopping and switching under conditions that one or two target(s) were presented at the beginning of each trial. Our findings show that when participants anticipate a stop signal, they delay initiating a movement in order to increase the chance to successfully stop the action. Interestingly, the results did not reveal this proactive inhibitory behavior when participants anticipate a switch signal, both when they were instructed to move towards a target location or when they were free to choose between two targets. Instead, they initiated faster movements similar to trials in which no adjustment in behavior was required - i.e., decision-making task without stop or switch signals.

By modeling this motor behavior within a recently developed neurocomputational theory [17], we predicted that the pause mechanism is engaged only when an outright stopping of action is anticipated. However, this action regulation mechanism fails to explain the faster responses to switch*ongoing* actions in instructed trials compared to choice trials, suggesting there might be a different mechanism for switching ongoing actions between instructed and choice movements. We considered an alternative hypothesis in which the pause mechanism is engaged to switch actions only in instructed trials. On the other hand, switching action in choice trials is implemented through a competition between the ongoing and the new actions. The rationale behind this hypothesis is that the neural activity associated with the new action has not been formed prior to the switch signal in instructed trials. Therefore, there might not be enough time for the new action to form and suppress the ongoing action without engaging a pause mechanism that further assists in the inhibition process. On the other hand, the new action (i.e., the action associated with the unselected target) has already been formed in the choice trials prior to the switch signal and therefore switching can be implemented on-time through the competition between the ongoing and the new action. This mechanism generates reaching movements with longer SRT in choice trials compared to instructed trials. Overall, this study advances our understanding of the action regulation mechanism, providing evidence that the involvement of the pause mechanism is not constant but rather selectively activated during specific phases of action switching.

### Computational approaches to action regulation

The complex mechanisms underlying action regulation have been investigated through diverse computational frameworks. Normative models, including drift diffusion models (DDM) [32] and race models [2,4,33], have been successful in explaining behavioral patterns in multiple decision-making tasks. The race model notably conceptualizes action inhibition as a competition between go and stop processes, similar to our implementation of the pause mechanism. However, these models typically abstract away from the underlying neural dynamics and focus on describing behavior at a computational level.

Mechanistic models, on the other hand, aim to explain how neural populations interact to produce behavior. Several biologically inspired frameworks have been developed, including recurrent circuit models [34], which demonstrate how attractor states emerge to represent categorical choices. These models incorporate recurrent synaptic excitation combined with slow cellular processes that enable temporal integration, along with feedback inhibition to implement competitive dynamics among potential options. Wong & Wang [35] demonstrated, through a simplified biophysically-based model, how neural circuits can achieve realistic decision-making times using NMDA receptor-dependent dynamics. These dynamics enable recurrent synaptic excitation, facilitating the temporal integration necessary for perceptual decision-making. The model shows how such circuits balance the excitatory and inhibitory processes to produce decisions within the time constraints observed in biological systems. Similar to our neurocomputational theory, DNF theory has been used to predict the neural mechanisms in a variety of cognitive and visuomotor tasks[12,36–38]. Schneegans et al. proposed a DNF-based learning model where visual working memory is facilitated through spatially distinct neural maps, offering insights into how a task might be learned by progressively binding features [38]. Similarly, Klaes et al. presented a DNF-based model showing that sensorimotor learning can bias decision-making by shifting neural dynamics, influencing how decisions are made [37]. Both the Schneegans and Klaes models suggest that learning alters neural processing to improve task execution, providing potential explanations for how tasks can be learned over time.

Although these studies have made significant contributions to understanding the neural and behavioral mechanisms underlying various cognitive and visuomotor tasks, few have simultaneously simulated both neural and behavioral aspects. Furthermore, none of these models provides an integrated framework for planning, executing, switching and stopping actions in dynamic environments. In contrast, our neurocomputational framework not only predicts both motor behavior and neural mechanisms in action regulation tasks, but also offers a unified model for how actions are planned, selected, and dynamically adjusted.

### Mapping computational framework components to brain regions

The computational framework presented in this study operates at a systems level, designed to qualitatively model and predict motor behavior and the underpinning neuronal activity patterns within neural ensembles. It is not intended to serve as a rigorous anatomical model and therefore, we avoid making strict associations between its components (e.g., individual dynamic neural fields or control schemes) and specific cortical or subcortical regions. Nonetheless, the framework effectively captures key features of neuronal activity observed across various brain areas involved in action regulation. The spatial sensory input field encodes the location of the targets and mimics the organization of the posterior parietal cortex [39,40]. The expected reward field aligns with the ventromedial prefrontal cortex (vmPFC) and orbitofrontal cortex (OFC), two key frontal areas involved in computing expected outcomes [41,42]. The reach planning field can be associated with the parietal reach region (PRR) [43,44] and the premotor dorsal cortex (PMd) [8,45], two cortical areas involved in planning of reaching movements. Additionally, the stop signal field can be equated to the right inferior frontal gyrus (rIFG), which is activated when a cue associated with response inhibition (i.e., stop signal cue) is detected [46,47]. Finally, the pause mechanism is implemented through the STN, which receives input from the rIFG when a stop signal is detected [48]. In the context of our study, the STN likely plays a crucial role in action switching, helping to inhibit ongoing actions to allow for the initiation of new responses.

### From Lashley’s hierarchies to affordance competition

The parallel preparation of actions, a concept central to our model, originates from Lashley’s influential 1951 work [49]. He argued against serial chaining theories, proposing instead that skilled behaviors are supported by the simultaneous activation of multiple potential actions. This idea has been confirmed in various domains, such as speech production, skilled typing, sequential movements and reaching movements[50–53]. Extending this framework, the affordance competition hypothesis posits that multiple actions are concurrently formed and compete over time, with the most suitable action selected based on evidence accumulation [20,21]. The behavioral findings in our study align with the concept of parallel action planning, where actions compete for selection. Specifically, participants showed longer reaction times when choosing between two targets compared to when reaching for a single target. Interestingly, they were better at stopping the action when choosing between two targets than when directed towards a specific target, regardless of the SSD. These findings support the action competition hypothesis, suggesting that competition among potential actions weakens their neuronal activation, making it more difficult for one action to surpass the activation threshold and initiate a movement in the choice trials. Therefore, inhibiting an action is easier in choice trials due to the weaker activation. In contrast, the lack of competition in the instructed trials lead to stronger neural activation of the reaching movement, resulting in faster reaction times but making it harder to inhibit the action.

### Switching mechanisms

The challenge of understanding how the brain switches between actions has been a longstanding focus of research. Studies by Georgopoulos and colleagues have provided key insights into this problem, highlighting mechanisms that allow the brain to dynamically adjust and select among competing motor plans [54–56]. Georgopoulos et al. showed that target location changes during reaching movements can be smoothly accommodated within normal reaction times [54]. Building on this behavioral evidence, Georgopoulos et al. identified corresponding neural adaptations in motor cortical activity during movement interruptions, providing direct evidence of the brain’s capacity to reorganize action plans [55]. Further, Massey et al. revealed that motor commands are prepared simultaneously during sequential tasks, supporting Lashley’s theory about parallel processing, where multiple actions are activated concurrently and dynamically adjusted during execution [56]. While these studies provide strong evidence supporting the parallel processing theory of motor actions, they leave open the possibility that selective inhibitory mechanisms might also be involved in suppressing specific movement plans. Our findings offer an alternative theory that incorporates the concept of parallel processing of actions but extends it further by proposing the involvement of an independent pause mechanism. This mechanism is context-dependent, varying particularly between instructed and choice conditions.

One of the main findings in our study is that people delayed initiating an action when expecting a stop signal both in instructed and choice trials. This RDE has been reported in previous studies [17,57,58] and has been associated with an “active braking mechanism” that increases the chance of abandoning a response in case stopping is required [59]. Notably, RDE was not found in the switch trials - i.e., participants did not proactively slow down their response when a switch signal was anticipated. This suggests that the pause mechanism is not engaged when switching of action is anticipated. However, when we modeled the switch task without the involvement of the pause mechanism, we predicted that it takes longer to switch a reaching movement in instructed than in choice trials. This is against the behavioral findings, in which instructed trials have shorter SRT than choice trials. To account for these results, we modeled the mechanism of switching action within a new architecture, in which the pause mechanism is engaged only in switching instructed trials. In this architecture, two inhibitory mechanisms are involved in switching actions: a) the pause mechanism that suppresses the current action and b) the inhibitory competition between the current and the new action. On the other hand, reaching movements in choice trials switch direction only through the inhibitory competition between the current action and the new action.

Therefore, a reasonable question is if instructed trials require a pause mechanism for switching ongoing reaches, why did people not exhibit a proactive planning behavior when they anticipated a switch signal, as they did when they anticipated a stop signal? One potential explanation is that the pause mechanism is not activated prior to movement initiation, since the goal is not to completely stop the ongoing action, but to reduce the neural activity associated with that action while the activity of the new one is formed. Another explanation is that switching of actions already involves an inhibitory mechanism - i.e., the inhibitory competition between the current and the new action. Therefore, the pause mechanism acts as an auxiliary mechanism to inhibit on-time the current action while the new one is formed. Another scenario that could account for the different SRTs between instructed and choice reaches is that when people decide between two targets, the decision process continues even after movement initiation as has been reported in “change-of-mind” studies [60,61] - i.e., initial decisions are revised after movement onset. Once the switch signal is cued, in the two-target switch trials, people might need to first stop the decision-making process and then re-direct their movements towards the new target location, which is an overall slower process than when they only need to re-direct their movements (i.e., one-target switch trials).

### Limitations, alternative hypotheses and future experimental studies

We must emphasize that the neurocomputational theory employed in this study is a systems-level theory designed to qualitatively predict motor behavior, and the underpinning neural mechanisms across the three experimental tasks. While the theory successfully captures many key features of motor behavior in action regulation tasks, it exhibits the inherent limitations of systems-level computational models that often cannot dissociate between alternative and competing hypotheses.

For instance, our model predicts a specific mechanism of action regulation to account for the longer SRT observed in choice trials compared to instructed trials. However, this explanation does not exclude alternative interpretations, such as those based on the capacity-sharing model. This model suggests that motor planning relies on finite processing resources distributed across competing movement plans [62–66] -i.e., the brain has limited resources (or capacity) that can be distributed across multiple tasks or motor actions. When planning multiple actions in the choice trials, shared processing resources are divided among the actions, potentially prolonging SRT due to the additional time required to redistribute these limited resources effectively across the various motor actions. Nonetheless, this theory does not preclude the involvement of a pause mechanism during resource reallocation.

Additionally, our joystick-based paradigm enables the exploration of computational principles underlying action regulation but offers limited insight into the biomechanical aspects of movement. Studies employing robotic exoskeletons and EMG recordings have provided detailed analyses of arm movements and muscle activation patterns during rapid online corrections and obstacle avoidance [67,68]. Future work integrating such biomechanical measurements with reward-based paradigms could bridge the gap between computational theories and motor system implementation, offering a more comprehensive understanding of action regulation.

While our neurocomputational framework is developed and validated for reaching tasks involving action selection, stopping, and switching, it can be extended to other motor behavior tasks (for more information see [16]). The interaction between action planning and inhibitory control through the reach planning field and pause mechanism captures fundamental computational processes in motor control. The model architecture can be adapted to diverse scenarios, such as reaching movements with goal location uncertainty [69], sequential reaching movements [70], eye-movements (i.e., saccades) [16], obstacle avoidance, and others. While the specific parameters governing DNF interactions and temporal dynamics would vary across these contexts, the core principles — i.e., competition between multiple action plans and context-dependent engagement of the pause mechanism — should remain applicable across the different tasks. Understanding how these parameters generalize across different motor contexts remains an important direction for future investigation.

Overall, the above considerations - ranging from alternative mechanisms to broader questions of how the framework generalizes to other motor control tasks - highlight both the limitations but also the strength of the framework to generate testable hypotheses. For instance, the presence or absence of a pause mechanism could be experimentally validated by recording neural activity from STN in humans or animals performing motor tasks that involve action switching. Similarly, simultaneous recording from both the motor cortices and STN could reveal how parallel processing and pause mechanisms might work together in action regulation. Such studies would provide critical insights into the neural underpinnings of action regulation and help refine the framework further.

### Conclusion

Overall, our study aims to better elucidate the computations underlying switching of actions and to assess whether switching is an extension of the stopping process, or it involves a different mechanism. To do so, we trained people to perform reaching movements in dynamic environments that involve stopping and switching of actions, and modeled their motor behavior within a neurocomputational framework. The results showed that action planning involves a pause mechanism only when a stop signal is anticipated. However, when a switch signal is anticipated, the pause mechanism is not engaged to delay movement initiation. Interestingly, the results suggest different mechanisms for switching actions when people are instructed to move towards a single target, and when they are free to choose between two targets. To conclude, our study provides novel insights into the computations of action regulations that involve action inhibition, opens new doors for further investigation of the action regulation mechanisms in neurophysiological studies.

## Materials and methods

### Ethics Statement

The study was approved by the University of California, Riverside Review Board and all individuals signed a written informed consent before participating.

### Participants

A total number of 20 neurologically healthy adults (9 females) participated in the study. The ages at the time of the experiment were 23.98 ± 4.88 (mean ± SD) years old. We determined the sample size for participants through a power analysis based on behavioral data (reaction time and probability of stopping an action) from our recently published study [17]. The analysis indicated that a total of 20 participants is sufficient to achieve statistically significant results (one-way ANOVA, p = 0.05 corrected for multiple comparisons, with an effect size of*f* = 0.4).

### Stimuli and experimental procedure

#### General.

All experiments were programmed using Psychophysics Toolbox Version 3 (PTB-3) for Matlab. Experimental setup is illustrated inFig 1. The participants sat in an experimental room approximately 60 cm from an LED monitor (Dell P2419HC). A two-dimensional joystick (Thrustmaster T.16000M FCS) was positioned in front of the sitting participants, with the base at the level of their elbows. The real-time position of the joystick was presented on the screen by a green circular cursor ( ~ 5.5 cm diameter). Participants were familiarized with the task by running a set of training trials, including reaches to one (instructed) and two (choice) target trials, both with and without stop and switch cues. Once they felt ready and comfortable with the tasks, the actual experiment started.

#### Decision-making task.

In the decision-making task, participants were free to choose between two targets by moving the cursor towards the selected target location. Choice trials were randomly interleaved with instructed trials, during which the participants had to reach towards a single target location (50% instructed and 50% choice trials). A trial started with the cursor appearing at the bottom center of the screen. After 1.0-1.1 s, a 5.5 cm diameter circular blue cue (instructed trial) or two blue cues (choice trial) were presented on the screen, indicating the location of the target(s). The targets were displayed at four possible locations on the screen, each positioned 20.37 cm from the joystick’s starting point. The targets were placed at angles of 0, 60, 120, and 180 degrees, making them equidistant and 60 degrees apart from one another. The participants were instructed not to move the joystick before the target(s) appear on the screen. Once the target(s) were presented, the participants had to move the cursor towards the instructed or to the chosen target location within 1.0 s. In the choice trials, the two targets were marked with a different number (5 or 10) that reflects the reward value. Although the two targets were assigned with different reward values, both options were regarded as “correct”. The reward difference was implemented to establish a consistent initial preference, ensuring that participants would typically select the higher-valued target, allowing us to systematically study switching behavior by removing this target. A reaching movement was considered successful if the cursor touched the target within 1.0 s after the presentation of the target(s). If the participants failed to reach the target within 1.0 s or initiate a movement prior to target(s) presentation, the trial was aborted. After each trial, the participant had to move the cursor back to the original starting position. Otherwise, they received a warning signal “please move the joystick back to the center to start the next trial”. The participants performed 2 blocks with 48 trials each (2 blocks x 48 trials = 96 total trials).

#### Stop signal task.

The stop signal task is similar to the decision-making task with the difference that participants had to completely stop their actions in a random subset of trials (33%). This proportion of stop trials was selected to balance unpredictability to ensure a sufficient number of events for robust statistical analysis. It consists of one-target stop task (i.e., instructed trials with stop signal) and two-target switch task (i.e., choice trials with stop signal) that were performed in separate blocks of trials. In the stop trials, the color of the target(s) turned red after a short delay (stop signal delay, SSD), signifying the immediate need to abandon the action. The participants were informed that stopping and reaching to target(s) are equally important. A trial started with the joystick cursor presented at the bottom of the screen. After 1.0-1.1 s, a single blue cue (instructed trial) or two blue cues associated with different reward values (choice trial) were presented on the screen, and the participants had to initiate a movement towards either the single target or the selected target location within 1.0 s. If the target(s) turned red, the participants had to abandon the movement immediately. This adaptive staircase procedure aims to maintain approximately 50*%* success rate in stop trials, preventing both ceiling and floor effects in performance. The participants performed 2 blocks of the one-target stop task - each block comprised 60 trials, of which 20 were stop trials (2 blocks x 60 trials = 120 total trials). They also performed 2 blocks of the two-target stop task - each block comprised of 72 trials, of which 24 were stop trials (2 blocks x 72 trials = 144 total trials).

#### Switch task in reward-based decisions.

The switch task was also similar to the stop signal task with the difference that participants had to perform corrected movements, instead of completely stopping their actions. It consists of one-target switch task (i.e., instructed trials with switch signal) and two-target switch task (i.e., choice trials with switch signal) that were performed in separate blocks of trials. In both tasks, the switch trials constitute a random 33*%* of the total trials. This proportion of switch trials was selected to balance unpredictability to ensure a sufficient number of events for robust statistical analysis. In one-target switch trials, the target was replaced after a short variable delay (named switch signal delay, SWSD) by a second target at a new location, prompting the participants to switch their actions towards the new target location. In two-target switch trials, the high-reward target was removed after SWSD, and the participants had to correct their actions by moving towards the remaining target (low-reward target). The participants were informed that switching and reaching are equally important. A trial started with the joystick cursor presented at the bottom of the screen. After 1.0-1.1 s, a single blue cue (instructed trial) or two blue cues associated with different reward values (choice trial) were presented on the screen, and the participants had to initiate a movement towards either the single target or the selected target location within 1.0 s. If switching action was prompted, an extra 1.0 s was given to the participants to complete their actions. Similar to the stop task, if the participants successfully switched to the new target without crossing the location of the old target, the trial was considered successful, the screen turned black and a new trial started. In this case, the SWSD increased by 50 ms, making the next switch trial more challenging. If the participants failed to switch to the new target (crossing the location of the old target or failed to arrive at the new target location), the trial was aborted and the SWSD decreased by 50 ms, making the next switch trial easier. This adaptive staircase procedure aims to maintain approximately 50*%* success rate in switch trials, preventing both ceiling and floor effects in performance. The participants performed 2 blocks of one-target switch tasks with 60 trials in each block - 40 instructed trials without switching and 20 one-target switch trials (2 blocks x 60 trials = 120 total trials). They also performed 2 blocks of two-target switch tasks with 72 trials in each block - 48 choice trials without switching and 24 two-target switch trials (2 blocks x 72 trials = 144 total trials).

#### Switch task in internally-guided decisions.

We are also interested in exploring whether the reward value affects the switching behavior. To do so, we recruited 6 participants to perform the switch task without assigning reward values to the targets. Instead, the participants performed “internally-guided decisions” - free choices that are not informed by any external contingencies. The switch signal was cued based on the traveled distance from the origin - i.e., “distance-threshold”. The distance-threshold was set to a random number between 1.2% to 3.2% of the total distance between the origin and the target(s) location. The switch task without reward also consists of one-target switch task and two-target switch task that were performed in separate blocks of trials. In one-target switch trials, the original target was replaced by a new target when the cursor exceeded the distance-threshold, prompting the participants to correct their actions towards the new target location. In two-target switch trials, the target that was closer to the cursor disappeared, prompting the participants to move towards the remaining target. Everything else was the same as the switch task in reward-based decisions. The participants performed 5 blocks of one-target switch task with 60 trials in each block - 40 instructed trials without switching and 20 one-target switch trials (5 blocks x 60 trials = 300 total trials). They also performed 5 blocks of two-target switch task with 72 trials in each block - 48 choice trials without switching and 24 two-target switch trials (5 blocks x 72 trials = 360 total trials).

### Statistical analysis

Cubic smoothing spline interpolation was used to smooth the joystick movement trajectories and to compute the velocity of the movements. RT was defined as the time between the target appearance and the time that movement velocity exceeded 10*%* of the maximum velocity within the trial. RTs faster than 100 ms were excluded from further analysis because anticipation is considered to be involved before participants initiate an action. RT outliers (RTs more than 3 standard deviations from the mean RT) were also excluded. RTs across all participants were pooled together, and two-way ANOVA analyses were performed using MATLAB’s anovan function with experimental task (3 levels: decision-making, stop, switch) and movement type (2 levels: instructed vs. choice) as fixed factors to determine the group differences in RTs. The function used Type III sum of squares to handle any unequal cell sizes. Post-hoc multiple comparisons were performed using the Tukey-Kramer method (via MATLAB’s multcompare function) following significant two-way ANOVA results. We also computed the reaction time for switching reaching movements (i.e., SRT) as the time between the switch signal (i.e., instructed/selected target disappears) and the time when the joystick starts to move towards the new target location. A two sample t-test was performed to compare the differences in SRT.

### Computational framework

We utilized a neurodynamical computational framework that was recently proposed by our group to model action regulation tasks that involve motor inhibition, such as selecting, stopping, and switching actions [17]. The computational framework is based on DNF theory and SOC theory, and uses a series of DNFs to model the neuronal circuitry for perception, expected outcome, effort cost, context signal, action planning, and execution. The functional properties of each DNF are determined by the lateral inhibition within the field and the connections with other fields in the architecture. The projections between the fields can be topologically organized – i.e., each neuron*i* in the field drives the activation at the corresponding neuron*i* in the other field, or unordered – i.e., each neuron in one field is connected with all neurons in the other field.

The architectural organization of the framework is shown inFig 4. The DNF platforms (except for the stop signal field and the pause field) consist of 181 neurons, with preferred direction between 0 and 180 degrees. The “spatial sensory input” field encodes the angular representation of the target(s), and the “expected outcome” field encodes the expected reward for reaching to a particular direction. The outputs of these two fields send excitatory projections (green arrows) to the “reach planning” field in a topological manner. The “reach cost” field encodes the effort cost required to implement a reaching movement at a given time and state. It sends inhibitory projections (red arrow) to the reach planning field to penalize high-effort actions. For instance, an action that requires changing of moving direction is more “costly” than an action of keeping going in the same direction. The “stop signal” field consists of 100 neurons and is activated when a stop cue signal (i.e., the color of the target turned red) is detected. It is linked via one-to-all excitatory projections with the pause field. The “pause field” is linked via one-to-all inhibitory connections with the reach planning field. Once a stop cue is detected, the pause field quickly suppresses the activity of the reach planning field to stop the planned or ongoing actions.

The normalized activity of the reach planning field describes the relative desirability$d_{i}(t)$ of each “reach neuron” with respect to the alternative neurons at time t – i.e., the higher the activity of a reach neuron*i*, the higher the desirability to move towards the preferred direction$\phi_{i}$ of this neuron with respect to the alternatives at a given time t. Each neuron*i* in the reach planning field is connected with an optimal control scheme that generates reaches. Once the activity of a particular neuron*i* exceeds an “action initiation threshold”, the controller is triggered and generates an optimal policy$\pi_{i}$ – i.e., a sequence of motor actions towards the preferred direction of the neuron*i*. Hence, a decision is made once a neuronal population exceeds the action initiation threshold and the performed action$\pi_{mix}$$\left(x_{t}\right)$ is given as a mixture of the active policies (i.e., policies with active reach neurons) weighted by relative desirability values of the corresponding neurons at any given time and state:

$$\begin{array}{c} \pi_{mix}(x_{t})=\sum_{j}^{j+M}d_{j}(x_{t})\pi_{j}(x_{t}) \end{array}$$ (1)

where$x_{t}$ is the state of the system at time t (i.e., position, velocity, acceleration, etc.),$d_{i}(t)$ is the normalized activity of the neuron*i*, and$\pi_{i}$ is the optimal policy generated by the controller connected with neuron*i*. Because desirability is time- and state-dependent, the weighted mixture of the individual policies$\pi_{mix}(x_{t})$ can be reprogrammed based on the new incoming information. For more details about the mathematics underlying the computational framework, see [16,17].

## Supporting information

S1 Table Modelparameters. The values of the neurocomputational model parameters used in the simulations.
(PDF)
