# Supplementary material for: Computational mechanism underlying switching of motor actions
Source: PLoS Comput Biol. 2025 Feb 10;21(2):e1012811. doi: 10.1371/journal.pcbi.1012811 (PMC11875378; doi:10.1371/journal.pcbi.1012811)
Supplement: S1 Table — The values of the neurocomputational model parameters used in the simulations. (PDF) [file pcbi.1012811.s001.pdf]

Model Parameters		
**Parameters**	**Description**	**Value**
η_{loc}	Visual input gain	8.5
η_{reward}	Expected outcome input gain	2.5
η_{cost}	Action cost input gain	-0.1
η_{pau}	Pause input gain	-4.0
γ	Action initiation threshold	0.6

Spatial sensory input field & Expected outcome field parameters		
**Parameters**	**Description**	**Value**
τ	Time constant	5.0
c_{exc}	Amplitude of excitatory portion of weight kernel	0
c_{inh}	Amplitude of inhibitory portion of weight kernel	0
σ_{exc}	Width of excitatory portion of weight kernel	5.0
σ_{inh}	Width of inhibitory portion of weight kernel	40.0
h	Resting activity level	-5.0
q	Noise level	0.25
σ_q	Width of noise kernel	5.0
β	Steepness of sigmoid activity function	1.0
g	Strength of global inhibition	0.8

Reach planning field parameters		
**Parameters**	**Description**	**Value**
τ	Time constant	5.0
c_{exc}	Amplitude of excitatory portion of weight kernel	20
c_{inh}	Amplitude of inhibitory portion of weight kernel	5
σ_{exc}	Width of excitatory portion of weight kernel	5.0
σ_{inh}	Width of inhibitory portion of weight kernel	180
h	Resting activity level	-5.0
q	Noise level	0.5
σ_q	Width of noise kernel	5.0
β	Steepness of sigmoid activity function	1.0
g	Strength of global inhibition	1

Pause field parameters		
**Parameters**	**Description**	**Value**
τ	Time constant	5.0
c_{exc}	Amplitude of excitatory portion of weight kernel	0
c_{inh}	Amplitude of inhibitory portion of weight kernel	0
σ_{exc}	Width of excitatory portion of weight kernel	5.0
σ_{inh}	Width of inhibitory portion of weight kernel	25.0
h	Resting activity level	-5.0
q	Noise level	0.25
σ_q	Width of noise kernel	5.0
β	Steepness of sigmoid activity function	1.0
g	Strength of global inhibition	0
